# Effect of Russian current expert modes on quadriceps muscle torque in healthy adults: A single-blinded randomized controlled trial

**DOI:** 10.1371/journal.pone.0297136

**Published:** 2024-01-25

**Authors:** Ansam Hasan, Ibrahim Moustafa, Tamer Shousha

**Affiliations:** 1 Department of Physiotherapy, College of Health sciences, University of Sharjah, Sharjah, UAE; 2 Neuromusculoskeletal Rehabilitation Research Group, Research Institute of Medical and Health Sciences, University of Sharjah, Sharjah, UAE; 3 Department of Basic Sciences, Faculty of Physical Therapy, Cairo University, Cairo, Egypt; 4 Department of Physical Therapy for Musculoskeletal Disorders and its Surgery, Faculty of Physical Therapy, Cairo University, Cairo, Egypt; 5 University of Sharjah Center of Excellence for Healthy Aging, Sharjah, UAE; 6 Healthy Aging, Longevity and Sustainability Research Group, Research Institute of Medical and Health Sciences, University of Sharjah, Sharjah, UAE; The Education University of Hong Kong, HONG KONG

## Abstract

**Background:**

Russian current (RC), a well-known neuromuscular electrical stimulation operating at 2500 Hz, has demonstrated significant strength improvement over traditional exercises due to its high tolerance and low pain provocation. Despite extensive NMES parameter research, the specific effects of expert modes, particularly ON2 and Rest, remain unexplored. This study investigates the direct effect of these expert modes on quadriceps muscle strength in healthy adults.

**Methods:**

This is a single-blind, randomization-controlled trial. Forty-eight healthy university students (31 females, 17 males) were assigned in two randomized experimental groups either the ON2 or Rest mode for a 15-minute electrical stimulation session. Quadriceps maximum voluntary isokinetic contraction measurements were taken before and directly after RC application using Biodex Medical Systems 4 pro isokinetic dynamometer.

**Results:**

Both RC modes significantly increased the quadriceps muscle torque in healthy adults compared to baseline (p<0.05). Baseline mean torque was 123.28 (SD = 38.8), and post- RC mean torque was 136.67 (SD = 45.76). Deviation from normality was observed at baseline (p = 0.034) and persisted post-RC application (p = 0.017). The Wilcoxon test reported significant increases in quadriceps muscle knee torque for both ON2 and Rest groups (p < 0.001). The lack of ties in ranks and negative Z-values highlight the robustness of the observed effects.

**Conclusion:**

The findings of this study align with previous research on NMES and RC supporting the idea that electrical stimulation enhances muscle strength, selecting the appropriate RC expert modes can assist physiotherapist in tailoring rehabilitation program to achieve their specific strength goals.

## 1. Introduction

Neuromuscular electrical stimulation is frequently employed for enhancing the strength of weakened muscle by utilizing electrical impulses to excite peripheral nerve, leading to contraction of muscle tissue [1–3]. Muscular strength improvement depends on the level of torque generated during the application of the stimulation [4, 5]. In 1977, Russian physiologist Yakov Kots reported a 30–40% increase in muscle strength among elite athletes after 20 sessions of using Russian current (RC) at a frequency of 2,500 Hz. This increase was attributed to the ability of RC to reduce skin resistance, facilitating deeper penetration to reach motor nerves at a greater depth. The outcome surpassed gains from exercise alone, as RC neuromuscular electrical stimulation (NMES) induced contractions 10% to 30% more powerful than those attainable through isometric contraction [6].

Similarly, numerous studies have been conducted to determine the RC parameters that are clinically adopted in generating stimulation that is efficient in its depth of penetration for both muscles and nerves to elect the greatest muscle torque including: electrode positioning [7], current frequency [6, 8–11], current amplitude [9, 12, 13], pulse duration [13, 14] and medium-frequency alternating current burst duty cycle [11, 15, 16],

Among the key parameters influencing the electrical stimulation efficacy, amplitude, which represents the intensity of the electrical stimulus. Higher burst frequencies sustained contraction, promoting muscular strength and endurance, low bust frequencies allow for muscular relaxation between stimuli making them suitable for intermittent contraction, pain management, and rehabilitation [6, 12, 13, 17].

Duty cycle refers to the ratio of time the stimulus is active to the total time including the rest period. The low duty cycle involves shorter active periods, allowing muscles to rest between stimulations. This can be useful for preventing fatigue during prolonged sessions. Conversely, a 100% duty cycle provides continuous stimulation, potentially inducing greater muscle fatigue but might be useful in specific therapeutic contexts. The choice of duty cycle depends on the desired outcome, considering factors like muscle recovery, fatigue management, and treatment goals in applications like rehabilitation or sports performance [6, 11, 16, 18, 19].

In electronics, the term "expert mode" refers to a specialized operating state or configuration within a device or system that provides unique functionalities or characteristics. The exact nature of a special mode can vary widely depending on the context and the specific electronic device or system in question [20]. In Russian current stimulation’s expert mode, user can either modulate the burst frequency and amplitude for the alternating subsequence burst, as seen in the ON2 mode, or modulate the inactive phase “off time” in Rest mode. This modulation introduces a burst during the off time with adjustable frequency and amplitude, consequently, change burst duration and the duty cycle.

Since various parameters of neuromuscular electrical stimulation (NMES) have been extensively studied, the specific effects of modulating burst frequency, amplitude, and duty cycle within the expert mode option of Russian current stimulation remain relatively unexplored in the existing literature, the study of the expert mode option in Russian current stimulation is crucial to advance our understanding of how modulating burst frequency, amplitude, and duty cycle within this specialized operating state can optimize neuromuscular electrical stimulation (NMES) outcomes.

Investigating the impact of expert modes of RC electrical stimulator on isokinetic quadriceps torque could provide valuable information for clinicians aiming to select optimal stimulation parameters. Hence, the objective of this study was to investigate the direct effect of Russian Current (RC), particularly ON2 and Rest modes, on the isokinetic torque of the quadriceps muscle and to determine if these two RC modes (ON2 and Rest modes) influenced quadriceps muscle strength in healthy adult participants. The outcome of this study may allow clinicians to anticipate the potential of these modes lies in their ability to generate a higher level of muscular training intensities at specified current amplitudes. The data regarding the impact of these modes on muscular training intensities could be of value for clinicians, aiding them in planning the most efficient NMES strength-training programs. Physiotherapist and clinicians can incorporate these findings in the development of electrical stimulation-based rehabilitation and muscle training program.

## 2. Methods

### 2.1 Study design and setting

A single-blind, randomized controlled trial. The study was prospectively registered at www.clinicaltrials.gov (NCT05303181), and approved by the Research ethical committee, University of Sharjah (REC-22-02-28-S). and the study was conducted in accordance with the standards set by the consolidated Standards of Reporting Trials (CONSORT). The trial was conducted in University of Sharjah physiotherapy laboratory, UAE. Recruitment of study started on January 14,2023 and ended on June 6,2023

### 2.2 participants

Forty-eight healthy adult university students consist of 31 females, 17 males, participated and successfully completed this study.

All study volunteers were recruited from the university community through a voluntary sign-up sheet displayed publicly and a mass e-mail distributed to all students. They had no history of surgeries on the lower dominant limb, hip, or knee pathogen, nor contraindications to neuromuscular electrical stimulation (NMES). Six participants had a left-dominant leg. The dominant limb was identified by asking the participant which leg they would use to kick a ball.

Before participating in this study, all participants signed a written consent form, including an agreement to participate and consent for the publication of the results. This study and its procedures were approved by the Research Ethics Committee of the University of Sharjah.

Participants were informed that the study aimed to investigate the concentric quadriceps muscle torque output generated after the application of various modes of RC electrical stimulation. A general medical questionnaire filled by the participant as a baseline assessment, which included information about their age, sex. BMI and history.

Before testing, participants’ blood pressure was measured at rest, following the guidelines set by the American College of Sports Medicine (ACSM) for exercise [21]. Participants were excluded if the resting blood pressure equaled or exceeded 180 mmHg systolic/110 mmHg diastolic. Prior to the actual testing, participants underwent 5 minutes warm up at low intensity on a cycle ergometer and preformed stretching exercises for the quadriceps muscle and hamstrings (3 repetitions of 30 seconds each) to reduce the risk of muscle strain during the test process [22].

All study participants were familiarized with the testing protocol that consisted of three voluntary maximum isokinetic knee contraction as a base line measurement, followed by a 15-minute session of RC electrical stimulation using one of expert modes to reduce any misunderstandings related to the test procedure.

### 2.3 Maximum Voluntary Isokinetic Contraction (MVIC) measurements

An Isokinetic dynamometer (Biodex Medical Systems 4 pro, Inc, Shirley, NY) was used to evaluate the torque production, and it was calibrated prior to the session began. For all testing, the dynamometer was adjusted to operate at a specific angular velocity of 60°/s (isokinetic mode) and the dominant limb was placed at a 90° angle of knee flexion [23, 24]. The dynamometer’s resistant lever arm was positioned slightly above the ankle malleoli to stabilize the lower leg, and the pivot point of the lever arm was adjusted to be align with the lateral epicondyle of the knee to prevent excessive movement of the trunk. The participant was securely fastened to the seat with three nonelastic belts: two diagonal chest straps from shoulder to the opposite hip and one waist strap encircling the hip at the level of the anterior superior iliac crests.

The highest knee extension torque produced by the participant was assessed using maximum voluntary Isokinetic contraction (MVIC) conducted while the knee at 90 degrees of flexion against the dynamometer resistance lever arm. After being fastened into Biodex machine and before the stimulation was applied, participants executed at least three maximum voluntary isokinetic contractions (MVIC), with a 90-second rest between each [23].

The highest peak torque measured in Newton meters (Nm), achieved among the three maximum-effort trials was recorded using the Biodex as a base line measurement. For instance, if there was a rise in the maximum torque on the third attempt exceeding 5%, participants were instructed to sustain their contraction efforts until the rise in maximum torque dropped to less than 5%. The examiner consistently provided verbal encouragement to the participants by the examiner to ensure they exerted maximal effort during all contractions.

### 2.4 Russian current experts’ modes

Neuromuscular electrical stimulation, particularly the RC stimulation, was delivered using a GymnaUniphy Combi200 stimulator (Bizlen, Belgium) with specific parameters. Carrier frequency was set at 2500 Hz, pulse time being 200 milliseconds and burst frequency of 50Hz with on time of 2 second followed by an off interval of 2 seconds, resulting in a 50% duty cycle as fixed parameter, which automatically adjust the expert modes: ON2 mode consists of two intervals: the stimulus applied as preset on the device with amplitude incrementally increased with the purpose of reaching the participants’ maximum tolerance 7/10 followed by a two second off period and then another ON2 stimulus with a frequency of 50 Hz and 100% amplitude then the Rest mode, which also consisted of two intervals: the stimulus applied as preset on the device, then followed by a continuous Rest stimulus that has a frequency of 4 Hz and 70% amplitude. Two channels were used for delivering the electrical stimulus, with four rectangular cutaneous electrodes (6×8 cm diameter) positioned on the anterior surface of the thigh, targeting the motor points of both vastus medialis and rectus femoris following a previous study done by Dantas et al. [25–27].

Participants were monitored and they were instructed to self-report any pain on an 11-point (0–10) numeric pain-rating scale after each contraction, with 0 indicating “no pain” and 10 representing the “worst pain imaginable” [28]. Participants were informed that a pain level of 7/10 was the maximal acceptable pain, and the trials were terminated directly once the pain rating reached 7/10 or if they wished to stop testing for any other reasons. Similarly, participants were instructed to stop stimulation if they experienced a similar level of discomfort.

Study participants were randomly assigned into two test groups and the knee extensor torque was measured in response to each mode based on the participants’ maximum tolerance. Participants were blinded to the type of expert mode they admitted with.

In the first testing, participants were admitted to RC electrical stimulation session with expert mode ON2. This mode consists of two intervals: the stimulus applied as preset on the device with amplitude incrementally increased with the purpose of reaching the participants’ maximum tolerance 7/10 followed by a two second off period and then another ON2 stimulus with a frequency of 50 Hz and 100% amplitude. While the second group received the session with Rest mode, which consisted of two intervals: the stimulus applied as preset on the device, then followed by a continuous Rest stimulus that has a frequency of 4 Hz and 70% amplitude. Each participant completed a single 15-minute session of RC, applied in a randomly selected expert mode to the dominant thigh.

### 2.5 Statistical analysis

The sample size was calculated using a non-published pilot study on 10 participants, five per group considering the quadriceps torque as a primary outcome. The non-published pilot study was designed and carried out by the authors to mimic the same context of this experiment with the same experimental conditions described in the methodology section later. The required sample size was calculated using the G-power software, by assuming 80% power with a significance level of 0.05, and effect size of 0.6, to detect the minimum mean difference of 15 points and a standard deviation of 25, the sample size required was 21 in each group. When considering a 10% dropout, the sample required for both groups was 24 [29, 30].

This study included a random assignment of volunteers into two groups.

Randomization and allocation were done by an independent therapist in the clinic blinded from the purpose and outcomes of the study.

**Fig 1:**
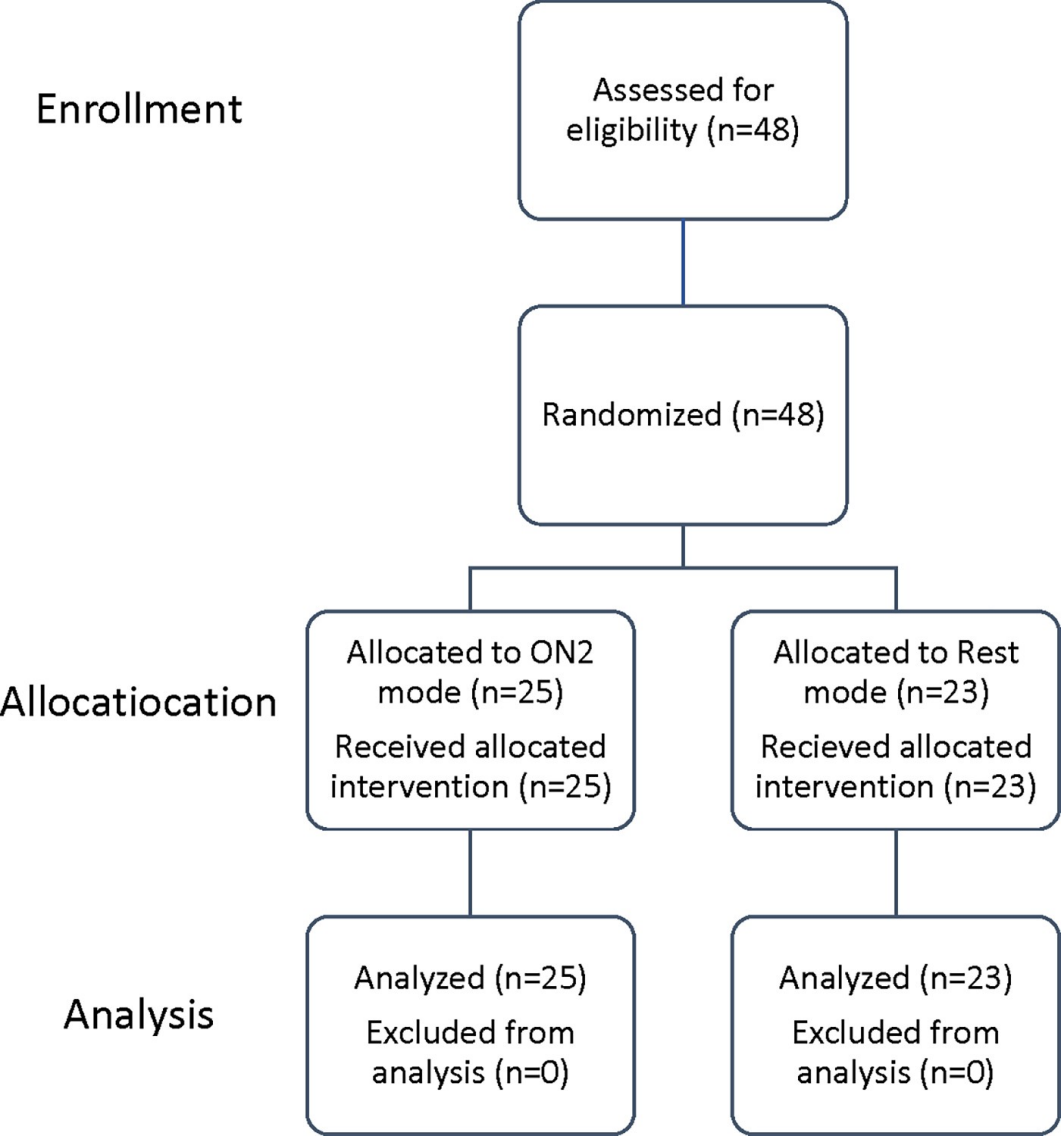
Flow chart of the RC study.

Randomization was limited to permuted blocks of different sizes (4,6 and 8) to ensure an equal number of allocations in each group. Each random block was stored in a locked drawer until required. They were stored as opaque sealed envelopes consecutively sequenced and numbered. The researcher opens the subsequent envelope after each participant formally enters the trial. Participants were blinded to the study hypotheses and remained unaware of which type of RC mode being used. The statistician was also blinded to intervention allocation; the data was coded in an unrecognizable manner, with no information indicating to which group a single participant had been assigned. However, the examiner was aware of the study due to the necessity for practice and expertise with the RC application [Fig 1].

The Maximum Voluntary Isokinetic knee extensor Contraction (MVIC) was measure using the Biodex system before the application of Russian current and directly after the session for two randomize groups.

Data analysis was conducted using Microsoft word EXCL (2016) as well as SPSS version 21.0 (IBM corporation, Armonk, NY). To assess the normality of the torque data, the Shapiro-Wilk test was employed. The Wilcoxon test was conducted to compare the pre and post torque following the application of RC without normal distribution. The significant for all testing was set at an alpha level (p) of 0.05.

## 3. Results

The data from 48 participants were collected and subsequently analyzed (refer to Table 1). The study aimed to investigate the direct effect of two RC expert modes on quadriceps muscle knee torque. The participant composition consisted of 31 females (64.6%) and 17 males (35.4%). In term of limb dominance, approximately 12.5% (6 participants) exhibited left dominance, while the majority 87.5% (42 participants), demonstrated right dominance.

**Table 1:** Participants’’ descriptive data.

Descriptive information for participants*	
Measure	Outcome
Age (y)	20.7±1.4(18–25)
Weight (Kg)	61.6±12.6(37–97)
Height (cm)	165.4±10.2(144–189)
BMI (kg/m^2^)	22.6±2.9(16.7–32)

*Total sample, n = 48. Values are mean ± SD

### 3.1 Knee torque measurements

The baseline and post-treatment measurements exhibit variation in central tendency and variability. The confidence intervals provide a range for the population means, and skewness and kurtosis inform about the shape of the distributions. Further analyses and comparisons were performed to assess the impact of the treatment and explore relationships within the data at baseline (Table 2).

**Table 2:** Analysis of quadriceps maximum torque measurements pre and post RC application.

Quadriceps maximum torque measurement pre and post RC application				
			Statistic	Std. Error
Pre RC application (Baseline measurement)	Mean		123.279	5.5994
	95% Confidence Interval for Mean	Lower Bound	112.015	
		Upper Bound	134.544	
	5% Trimmed Mean		121.994	
	Median		117.900	
	Variance		1504.979	
	Std. Deviation		38.7941	
	Minimum		63.2	
	Maximum		208.0	
	Range		144.8	
	Interquartile Range		59.8	
	Skewness		0.558	0.343
	Kurtosis		-0.554	0.674
Post RC application	Mean		136.873	6.6050
	95% Confidence Interval for Mean	Lower Bound	123.585	
		Upper Bound	150.160	
	5% Trimmed Mean		135.256	
	Median		127.850	
	Variance		2094.034	
	Std. Deviation		45.7606	
	Minimum		69.1	
	Maximum		243.1	
	Range		174.0	
	Interquartile Range		80.1	
	Skewness		0.535	0.343
	Kurtosis		-0.765	0.674

**Fig 2:**
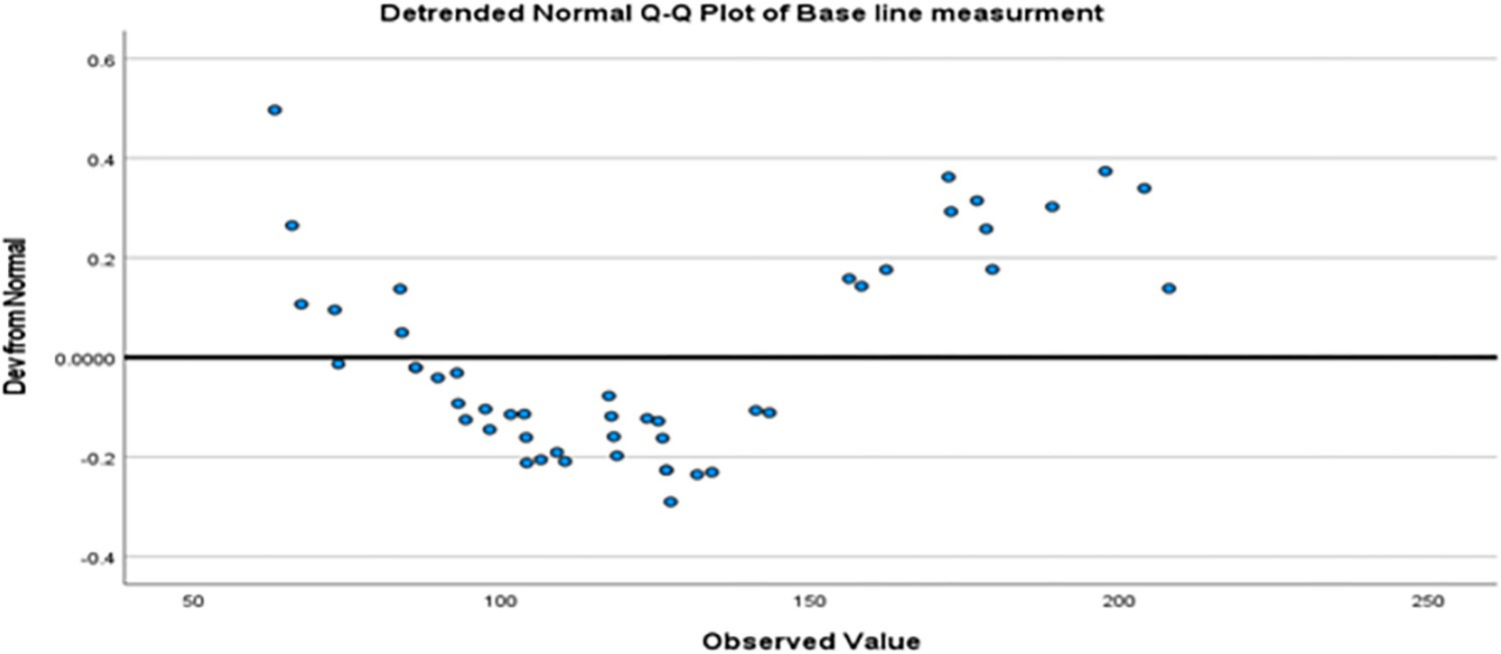
Quadriceps muscle knee torque measurements at the baseline.

Normality of the data set was analyzed by Shapiro-Wilk test. For baseline values, the Shapiro-Wilk test revealed a p-value of 0.034, suggesting a substantial departure from normality at the 0.05 significance level. Despite its statistical importance, the Q-Q plot visually suggests a relatively close alignment of data points with the theoretical normal distribution [Fig 2].

**Fig 3:**
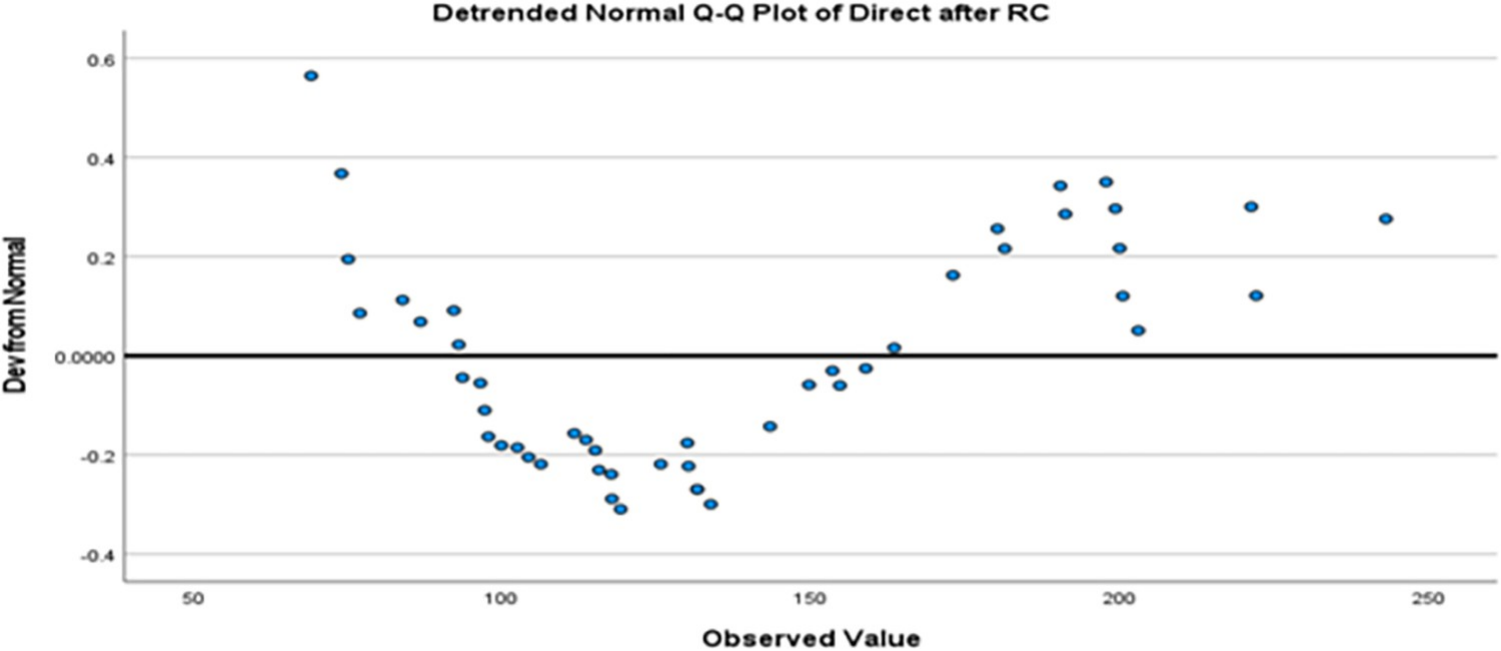
The quadriceps muscle knee torque measurement’s directly after the application of the RC expert modes.

While measurements obtained directly after RC, the Shapiro-Wilk test resulted in a p-value of 0.017, suggesting a significant departure from normality. The Q-Q plot supports this finding, revealing deviations from the straight line expected for a normal distribution [Fig 3].

To assess the direct effect of the RC expert’s modes intervention on muscle knee torque, Wilcoxon test was conducted. Wilcoxon Signed Ranks Test results indicate significant differences in quadriceps muscle knee torque between pre and post RC application for both the ON2 and Rest groups upon the use of Russian current p value < 0.05. There were 45 cases out of 48 with a post peak torque higher than baseline measurement. In the ON2 group, the mean baseline score was 121.112, which significantly increased to 135.028 in post RC session (Z = -4.292, p < 0.001). Similarly, in the Rest group, the baseline mean of 125.635 showed a significant rise to 138.878 in the post RC condition (Z = -4.046, p < 0.001). The negative Z-values for both groups suggest that post RC scores were consistently higher than the baseline scores (Table 3).

**Table 3:** Intragroup analysis for pre and post maximum torque measurements.

Variable	RC (ON2) n = 25			RC (Rest) n = 23		
	pre	post	z	pre	post	z
Peak torque	66±197.7	69±222.1		63.2±208	75.1±243.1	
Mean ±SD	121.1±38.7	135±45.6	-4.292*	125.6±39.7	138.9±46.9	-4.046*

Abbreviations: RC, Russian Current; SD, standard deviation

*P<0.05, Wilcoxon signed Ranks Test, Based on negative ranks

These findings highlight the effectiveness of the two RC expert modes in eliciting increased quadriceps muscle knee torque in both groups. The absence of ties in the ranks and the low p-values underscore the robustness of these observed effects.

## Discussion

This study investigated the direct effect of NMES, Russian Current (RC), on isokinetic concentric quadriceps muscle torque. This study’s main aim was to investigate if these two RC modes, ON2 and Rest modes, affected quadriceps muscle strength in healthy adult participants. The result of this study found that both RC modes, ON2 and Rest modes, led to significant increase in isokinetic knee extensor torque when compared to baseline measurement.

The finding of this study agrees with previous research on the use of NMES, particularly the RC, in enhancing the muscle strength [6, 31, 32], consistent with the principle of electrical stimulation and muscle recruitment; motor unit recruitment during voluntary contraction follows the size principle described by Henneman, Milner-Brown, and Jabre and Spellman [33]. According to this principle, small motor units (with type I skeletal muscle fibers) are recruited first, followed by the large motor units (with type II skeletal muscle fibers) as the force demand increases during voluntary contraction [34]. The order of recruitment of type II fibers occurs in the following order: first type IIa fibers, and then type IIb fibers. It has been suggested that NMES leads to an inversion of the size principle, recruiting larger (fast) motor units before the slow. This theory is based on two well-established findings: larger motor unit’s axons have a lower threshold for excitability, and data show increased fatigue in the NMES compared to voluntary activation [35, 36]. Theoretically, voluntary muscle contraction that is enhanced by electrical stimulation should result in a more powerful muscle contraction due to an increase in force production and additional muscle fiber recruitment in acute application of electrical stimulation and subsequently improve muscle power, strength, and endurance in chronic application [34, 37]. Therefore, the use of RC different modes, in this case, ON2 and Rest modes, affected the recruitment pattern of motor units contributing to the observed increase in knee torque.

While some studies suggest that electrical stimulation and exercise yield similar effects [38], Aspinar et al. assert that relying solely on electrical stimulation is inadequate for muscle strength enhancement. They emphasize the necessity of incorporating training programs with voluntary muscle activation in rehabilitation. However, a recent study examined the efficacy of three distinct methods—High Voltage Pulsed Galvanic Current, Russian currents, and isometric exercise—in enhancing isometric muscle strength in healthy women. The results demonstrated the effectiveness of all three methods without any observed superiority among them [39].

Selecting the “expert mode” option in Russian current stimulation allows to either modulate the burst frequency and amplitude for the alternating subsequence burst, as seen in the ON2 mode, or modulate the inactive phase “off time” in Rest mode. This modulation introduces a burst during the off time with adjustable frequency and amplitude, consequently, change burst duration and the duty cycle reference.

A significant issue connected with the use of NMES is the lack of well-established, evidence-based standards for optimizing the production of generated torque [40]. Ensuring maximum torque output is critical, especially when the goal of therapy is muscle growth and expansion. Several clinical studies have shown that raising the frequency and amplitude of the electrical current is limited by the rapid onset of muscular fatigue and the individual’s tolerance to the given current cycle [12, 15, 17]. Although RC was recommended by most studies, revealing an increase in the isometric induced torque, no study investigated the effect of RC on the maximum quadriceps isokinetic knee torque [41]. The current study used Russian parameters of 50-burst- per second while the amplitude increased to maximum tolerance by the participant with a pulse time of (200ms), set with on time 2s and off time 2 s.

Binder-Macleod et al. conducted experiments on the quadriceps femoris muscles in 1995 to investigate the effects of stimulation intensity on the physiological responses of human motor units. The researchers compared force-frequency relationships at various stimulation intensities, as well as responses to electrically elicited fatigue tests at various frequencies and intensities. The force-frequency relationship between 20% and 50% of maximum voluntary isometric contraction (MVC) showed no significant differences, with only a slight shift observed at 80% MVC. Fatigue was greater at 50% MVC, and fatigue increased with higher frequency within each force level. According to the findings, the physiological recruitment order during transcutaneous electrical stimulation may be less orderly than previously thought [12]. Word et al. reported that evaluation of electrical stimulation depends on discomfort and muscle torque, varying with frequency and burst duration. The study suggested a short burst duration (2–4 millisecond) of alternating current at customized frequencies for improved torque and comfort challenging the conventional practice and benefit in field [41, 42].

In 2009, a study done by Kesar et al. compared three strategies used during functional electrical stimulation (FES) to see how they affected muscle force output and fatigue. Constant pulse-duration with stepwise frequency increases (frequency-modulation), constant frequency with stepwise pulse-duration increases (pulse-duration-modulation), and constant frequency and pulse-duration (no-modulation) were among the strategies used. The results indicated that frequency-modulation outperformed pulse-duration-modulation in both peak forces and force-time integrals during fatiguing trains, while inducing similar levels of muscle fatigue. Despite frequency-modulation not being widely used in FES, clinicians should consider this strategy to enhance muscle performance [17].

Taking the previous study into consideration, the ON2 mode was adjusted to give the same stimulus frequency and amplitude as the ON stimulus (first stimulus) as the current study did not include a control group. Similarly, the Rest mode was adjusted to provide the same ON stimulus, while the Rest burst adjusted at frequency of 4 burst with amplitude of 70% of the ON stimulus, modulating the frequency and duration in light of a previous study suggesting that reducing frequency has an effect on muscle activity, as demonstrated by Gorgey et al. [13] on neuromuscular electrical stimulation (NMES), varying current amplitude and pulse duration showed no influence on muscle fatigue. However, reducing stimulation frequency from 100 to 25 Hz significantly decreased fatigue from 76% to 39%. Torque per active area explained 57% of fatigue variability between different protocols. These findings suggest that modifying stimulation frequency may be a key factor in mitigating muscle fatigue during NMES [42]. On the other hand, Gorgey et al. [14] investigated the impact of pulse duration and stimulation duration on evoked knee torque while accounting for the activated area through MRI. The study revealed longer pulse duration led to significantly higher torque output than shorter pulse duration but not stimulation duration. Other studies done by Bickel et al and Gregory et al. [43] matching initial torque with different stimulation parameters influences skeletal muscle fatigue. The investigation concluded that frequency is the main regulator for muscle fatigue, but NMES parameters could enhance the muscle torque generation without increasing the level of fatigue. Therefore, they recommended a protocol with low frequencies and longer pulse duration.

The authors believe that understanding how different Russian current modes affect the muscle torque can help therapist tailor rehabilitation protocol to achieve specific strength goals; if the patient case required a rapid strength gain, ON2 mode, with higher frequency and amplitude could be more useful. On the other hand, Rest mode with lower frequency and amplitude could be used for patients who need a gradual protocol of muscle strengthening.

In a recent study comparing voluntary isometric contraction (IC), Russian current (RC), and superimposed RC onto IC (SRC) for quadriceps strength and lower-extremity endurance found that RC and SRC outperformed voluntary IC in strength, with SRC outperforming RC in endurance [44]. These findings are consistent with previous research on neuromuscular electrical stimulation (NMES). In relation to this, our research lays the groundwork for potential advances in NMES precision via the ’expert mode’ in RC stimulation, allowing for nuanced adjustments for personalized interventions. While our research provides a foundation, investigating the potential of the expert mode offers a promising avenue for tailoring neuromuscular outcomes.

## Limitations

The current study has several limitations. The hamstring muscle group was not explicitly observed during this procedure, and it is possible that the net torque of the knee extension may decrease due to the hamstrings contracting. However, the anterior thigh’s tissue thickness makes it extremely unlikely that the current would have reached the hamstring muscles. Another limitation of this study was this trial was only performed on healthy volunteers’ quadriceps femoris muscles without control group. Last, as the distribution did not consider the homogeneity of characteristics, the authors noticed a difference between the standard deviations reported from the pilot study and the deviations reported from the main study. The discrepancy might be due to the gender or age differences between both studies.

Therefore, future research is recommended to determine whether the results of the current study can be generalized to other muscle groups or populations.

## Conclusion

This study advances our knowledge of how different expert modes of RC electrical stimulation affect quadriceps muscle torque. The result indicates that both expert modes, ON2 and Rest modes, can result in significant increase in the quadriceps muscle torque compared to baseline measurements. Physiotherapists and clinicians can utilize these findings when developing electrical stimulation-based rehabilitation and muscle training programs. More future research is recommended to confirm these findings in larger and more varied populations, also to evaluate the long-term effect of the expert modes on muscle strength. We also recommend to replicate the study without the prior testing exercises to detect the sole effect of Russian currents on the Quadriceps peak torque.

## Supporting information

S1 Checklist CONSORT statement. (DOC)

S1 File (PDF)
