# Supplementary material for: Effect of Russian current expert modes on quadriceps muscle torque in healthy adults: A single-blinded randomized controlled trial
Source: PLoS One. 2024 Jan 25;19(1):e0297136. doi: 10.1371/journal.pone.0297136 (PMC10810422; doi:10.1371/journal.pone.0297136)
Supplement: S1 Checklist — (DOC) [file pone.0297136.s001.doc]

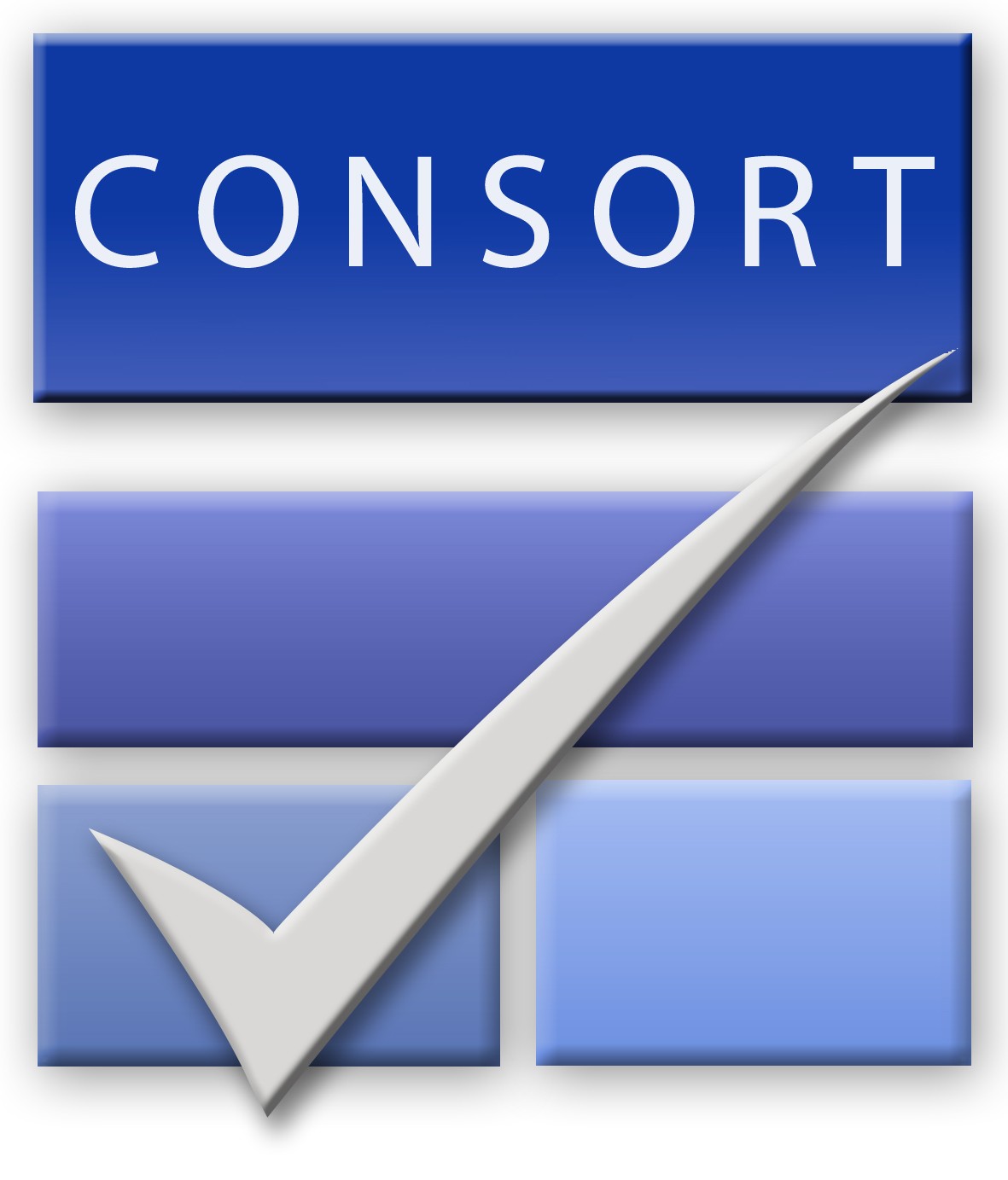
CONSORT 2010 checklist of information to include when reporting a randomised trial*

| Section/Topic | Item No | Checklist item | Reported on page No | Comments |
| --- | --- | --- | --- | --- |
| Title and abstract | | | |  |
|  | 1a | Identification as a randomised trial in the title | Yes p1 |  |
|  | 1b | Structured summary of trial design, methods, results, and conclusions (for specific guidance see CONSORT for abstracts) | Yes P 2-3 |  |
| Introduction | | | |  |
| Background and objectives | 2a | Scientific background and explanation of rationale | Yes-p 3-4-5 | Succeed to justify the need of study. |
|  | 2b | Specific objectives or hypotheses | Yes p 4-5 | Hypotheses were described clearly in the last introduction paragraph. |
| Methods | | | |  |
| Trial design | 3a | Description of trial design (such as parallel, factorial) including allocation ratio | Yes-p5 |  |
|  | 3b | Important changes to methods after trial commencement (such as eligibility criteria), with reasons | NA | No reported changes in the methods |
| Participants | 4a | Eligibility criteria for participants | Yes –p6 | Fully descriptive for inclusion and excluding participant |
|  | 4b | Settings and locations where the data were collected | Yes-p5 | First paragraph of the method |
| Interventions | 5 | The interventions for each group with sufficient details to allow replication, including how and when they were actually administered | Yes-p7-8-9 |  |
| Outcomes | 6a | Completely defined pre-specified primary and secondary outcome measures, including how and when they were assessed | Yes p5 | No secondary outcomes reported |
|  | 6b | Any changes to trial outcomes after the trial commenced, with reasons | NA | No changes required |
| Sample size | 7a | How sample size was determined | Yes-p9 | Determined by pilot study |
|  | 7b | When applicable, explanation of any interim analyses and stopping guidelines | NA |  |
| Randomisation: |  |  |  |  |
| Sequence generation | 8a | Method used to generate the random allocation sequence | Yes –p10 | Permuted blocks of different sizes |
|  | 8b | Type of randomisation; details of any restriction (such as blocking and block size) | P 10 |  |
| Allocation concealment mechanism | 9 | Mechanism used to implement the random allocation sequence (such as sequentially numbered containers), describing any steps taken to conceal the sequence until interventions were assigned | P10 | Reported |
| Implementation | 10 | Who generated the random allocation sequence, who enrolled participants, and who assigned participants to interventions | P10 | Reported |
| Blinding | 11a | If done, who was blinded after assignment to interventions (for example, participants, care providers, those assessing outcomes) and how | Yes- p10 | Reported |
|  | 11b | If relevant, description of the similarity of interventions | NA |  |
| Statistical methods | 12a | Statistical methods used to compare groups for primary and secondary outcomes | Yes- p10 | Reported |
|  | 12b | Methods for additional analyses, such as subgroup analyses and adjusted analyses | NA |  |
| Results | | | |  |
| Participant flow (a diagram is strongly recommended) | 13a | For each group, the numbers of participants who were randomly assigned, received intended treatment, and were analysed for the primary outcome | Yes –p11 | Describe the number of participants |
|  | 13b | For each group, losses, and exclusions after randomisation, together with reasons | Yes –p11 | Reported |
| Recruitment | 14a | Dates defining the periods of recruitment and follow-up | Yes –p6 | Reported |
|  | 14b | Why the trial ended or was stopped | NA | Not reported |
| Baseline data | 15 | A table showing baseline demographic and clinical characteristics for each group | Yes –p11 | Reported |
| Numbers analysed | 16 | For each group, number of participants (denominator) included in each analysis and whether the analysis was by original assigned groups | Yes –p 9-11 | Mentioned in results section |
| Outcomes and estimation | 17a | For each primary and secondary outcome, results for each group, and the estimated effect size and its precision (such as 95% confidence interval) | Yes- p11 | Reported |
|  | 17b | For binary outcomes, presentation of both absolute and relative effect sizes is recommended | NA |  |
| Ancillary analyses | 18 | Results of any other analyses performed, including subgroup analyses and adjusted analyses, distinguishing pre-specified from exploratory | NA |  |
| Harms | 19 | All important harms or unintended effects in each group (for specific guidance see CONSORT for harms) | Yes –p9 | Reported |
| Discussion | | | |  |
| Limitations | 20 | Trial limitations, addressing sources of potential bias, imprecision, and, if relevant, multiplicity of analyses | Yes-p15 |  |
| Generalisability | 21 | Generalisability (external validity, applicability) of the trial findings | Yes -p15 | Conclusion |
| Interpretation | 22 | Interpretation consistent with results, balancing benefits, and harms, and considering other relevant evidence | Yes –p12-15 | Discussion with other evidence study was mentions |
| Other information | | |  |  |
| Registration | 23 | Registration number and name of trial registry | Yes-p5 | Reported |
| Protocol | 24 | Where the full trial protocol can be accessed, if available | Yes- P5 | www.clinicaltrials.gov (NCT05303181) |
| Funding | 25 | Sources of funding and other support (such as supply of drugs), role of funders | NA | No funding |

see [www.consort-statement.org](http://www.consort-statement.org/).
