# Supplementary material for: Effect of Russian current expert modes on quadriceps muscle torque in healthy adults: A single-blinded randomized controlled trial
Source: PLoS One. 2024 Jan 25;19(1):e0297136. doi: 10.1371/journal.pone.0297136 (PMC10810422; doi:10.1371/journal.pone.0297136)
Supplement: S1 File — (PDF) [file pone.0297136.s002.pdf]

The effect of different Russian Current parameters on maximum voluntary isometric contraction of the quadriceps muscle among University of Sharjah students: A study protocol for a randomized controlled trial

Abstract

Background: Neuromuscular electrical stimulation (NMES) is widely used in rehabilitation that stimulates the peripheral nerve to cause visible muscle contraction to strengthen weak muscles. Physiotherapists utilized a variety of electro-motor stimulation modalities to enhance muscle performance, muscle reeducation, decrease muscle spasticity, and postpone atrophy. One of the most well-known neuromuscular electrical stimulations is the Russian current, recommended for boosting muscle strength due to its high tolerance and low pain provocation. As individual NMES parameters have not been clearly evaluated in literature, independent evaluation of individual NMES parameters can provide clinically relevant information on how NMES might be optimized to promote strength muscle training in both healthy and afflicted subjects. Of interest, an example of a parameter that has not received significant attention is the expert mode.

Methods

This is a single-blinded, block-randomization controlled trial in a prospectively controlled laboratory. Sixty-six healthy university students will be recruited from the University of Sharjah, United Arab Emirates (UAE). The volunteers will be divided into three systemically randomized experimental groups. Each group will be admitted to a program of training utilizing one of three expert modes of the Russian current. The electrically elicited quadriceps muscle extensor torque

will be measured in response to modes at the participants' maximum tolerance at three points before the start of training, after three weeks of training, and at the six-week follow-up by the Biodex isokinetic system.

Hypothesis

We hypothesize that electrically induced torque of the quadriceps muscle in healthy persons will be differently affected by various Russian current modes.

Impact

Russian current parameters have previously been researched, including phase duration, pulse duration, duty cycle, carrier frequency, and waveform type. However, there are no studies that have investigated how expert modes affect the quadriceps muscle's maximum voluntary isometric contraction.

Trial registration

The study is officially registered at www.clinicaltrials.gov (NCT05303181). Date 21/3/2022, and approved by the Research Ethics Committee, University of Sharjah (REC-22-02-28-S). Date 28/2/2022

1.Introduction

Neuromuscular electrical stimulation (NMES) is a type of physical therapy modality that uses electrical pulses to strengthen weakened muscles, which stimulate the peripheral neurons and result in contraction of muscle tissue. The amount of torque generated throughout the stimulation application is used to evaluate strength gains. NMES has been promoted as being more beneficial than exercise alone during the early phases of recovery. It has been well established in the literature that NMES can increase quadriceps femoris muscle strength; patients admitted to NMES training for 4 to 5 weeks at 3 sessions per week, had their quadriceps femoris muscle strength increased by about 14.0% to 48.5%. [1-7]. Multiple factors such as the well-being of the patient, position of the electrodes, frequency and current intensity, waveform, burst frequency, and duration encouraged researchers to investigate the best parameters to enhance muscle performance and minimize the discomforting because of the use of NMES.

Selkowitz et al. observed a 44% increase in quadriceps femoris muscle strength when training at an intensity of 91% of the maximum voluntary isometric contraction (MVIC) force.

On the other hand, according to Lai et al., investigating the effects of NMES training at 25% and 50% of the maximal voluntary isometric contraction, there was a corresponding increase in quadriceps femoris isometric strength of 24.2% and 48.5% [7]. Furthermore, Snyder-Mackler et al. [6] reported that after anterior cruciate ligament restoration, the intensity of NMES training was found to be positively correlated (r = 0.756, P 0.05) with the recovery of quadriceps femoris muscle strength. These investigations proved that increasing the intensity of the training stimulus (%MVIC) influenced muscle strength following NMES.

Motor unit recruitment during voluntary contraction follows the size principle as described by Henneman, Milner-Brown, and Jabre and Spellman [8]. According to this principle, small motor

units (with type I skeletal muscle fibers) are recruited first, followed by the large motor units (with type II skeletal muscle fibers) as the force demand increases during voluntary contraction [9]. Moreover, the order of recruitment of type II fibers occurs in the following order: first type IIa fibers, and then type IIb fibers. However, it has been suggested that NMES leads to an inversion of the size principle , in which recruiting larger (fast) motor units before the slow. This theory is based on two well-established findings: A larger motor unit's axons have a lower threshold for excitability, and data show increased fatigue in the NMES compared to voluntary activation [10, 11]. Theoretically, voluntary muscle contraction that is enhanced by electrical stimulation should result in a more powerful muscle contraction due to an increase in force production due to additional muscle fiber recruitment in acute application of electrical stimulation and subsequently improve muscle power, strength, and endurance in chronic application [9, 12]. A later study reported that voluntary muscle contraction and electrical stimulation causes different actions in muscle fiber and metabolism [13].

"What are the optimal stimulus parameters for eliciting forceful, yet comfortable, electrically induced muscle contractions?" To answer this question, a lot of research has been done to find which parameters are clinically adopted in producing depth-efficient stimulation of nerve and muscle. Therefore, selecting the best waveforms and time modulations to maximize muscle torque is a critical clinical factor. Laufer et al. [4] assessed which waveforms and current produced the greatest electrically induced torque (EIT) and minimal fatigue in the quadriceps femoris muscles. The study concluded that a sinusoidal waveform of 2500-Hz alternating current that was 50% burst modulated generated more fatigue and smaller EITs [% of maximum voluntary isometric contraction (MVIC)] compared to a biphasic rectangular symmetrical pulsed current. Using a 2500-Hz alternating current, McLoda and Carmack [14] studied the effects of various burst duty

cycles on the EIT (%MVIC) of the quadriceps femoris muscles. They found that the EIT was greatest at a burst duty cycle of 10% and the least at 90%. This implies that a lower duty cycle on time results in a higher EIT.

According to Snyder-Mackler et al. [15], stimulation of the quadriceps femoris muscle group with the 2500-Hz alternating current (P.01) or the symmetrical rectangular pulsed current (P.05) resulted in stronger EITs than stimulation with the 4000-Hz.

Similarly, Ward and Robertson [16] found that the EIT of the wrist extensor muscles decreased linearly as the alternating current carrier frequencies were raised from 1000 to 15000 Hz. According to the findings of these two previous studies, it might be possible to enhance the amount of EIT and reduce the current amplitude necessary to produce the greatest EIT response by utilizing burst-modulated alternating current with a lower carrier frequency and longer phase length.

Russian currents are medium frequency burst alternating currents with a carrier frequency of 2500 Hz and one of the most well-known neuromuscular electrical stimulations. A Russian physiologist [18] reported that using a frequency of 2.500 Hz; increased the muscle strength of the healthy subjects by 30-40 % in 20 sessions. Each session consists of 10 contractions in 10 seconds, each with 50 seconds' rest [17] which is considered a highly recommended protocol (10/50/10); where the stimulation to trigger muscle contraction is applied for 10 seconds, followed by off time for 50 seconds, and this cycle is continuously repeated 10 times. [19-21]

Expert modes are an option that can be selected after setting the electrical stimulator parameters, for example: carrier frequency, duty cycle, and burst duration. This option allows the therapist to modulate the stimulus in three diverse ways.

ON2mode; consists of two intervals the stimulus will be applied as; preset on the device, following by 2 second off and then and ON2 stimulus that has a frequency of 50 Hz and 100% amplitude.

Rest mode: consist of two intervals the stimulus will be applied as; preset on the device, then follow by a continuous Rest stimulus that has a frequency of 24 Hz and 83% amplitude.

Although, there has been a many studied that investigate the impact of several parameters on the quadriceps muscle's EIT, but no research demonstrates the relationship between expert modes and the increase electrically induced isometric quadriceps torque during the application of burst modulated alternating current at a specific peak current intensity. Since NMES is widely used to strengthen the quadriceps muscle, the impact of expert RC electrical stimulator modes on electrically induced quadriceps forces may offer further data to assist physicians in choosing the best stimulation parameters. Accordingly, this study's aim is to determine the electrically induced torque responses of the quadriceps to three expert types of burst-modulated alternating current at a particular peak current intensity. The results of this study could aid practitioners in using various parameters to improve muscle training at current amplitudes. With understanding of how different modes affect the intensity of muscular training, the physician could be able to provide the most effective NMES strength-training program.

2. Methods

2.1 Elaboration protocol

The protocol for the study was created in accordance with the SPIRIT guidelines [22] and is also reported in accordance with the CONSORT statement [23]. Figure 1 illustrates the schedule of assessments at various points in time.

2.2 Study design

A single blinded randomize controlled trial.

	Study period				
	**Enrollment**	**Allocation**	**Intervention**	**Post Intervention**	**Follow -up**
**TIMEPOINT**	-t1	0	Three weeks, 2x/week	3rd week (t1)	6th week (t2)
**ENROLMENT:**					
Eligibility screen	x				
Informed consent	x				
Allocation		x			
**INTERVENTION:**					
OFF-MOOD (RC)			←→		
ON-MOOD			←→		
ON2-MOOD			←→		
**ASSESSMENT:**					
List base line variable		x			
List outcome variable				x	x

FIGURE 1. SPIRT SCHEDULE OF ENROLLMENT, INTERVENTION, AND ASSESSMENT. THIS LIST INCLUDES THREE TIME POINTS. WHICH ARE BEFORE THE START OF INTERVENTION T0, AT THE END OF RC TRAINING T1, AND AT FOLLOW UP T2.

2.3 Study setting and eligibility criteria

This research will be conducted in the University of Sharjah physiotherapy laboratory, UAE. Healthy volunteer subjects of both sexes will participate in this study. The participants will be chosen from among university students via an outside posted volunteer sign-up sheet and a mass e-mail sent to all students. Before participation, all participants will sign a written consent

form that includes agreement to participation and publication of the results, as approved by the Research Ethics Committee of the University of Sharjah. The participant will be informed that the study's purpose is to measure the output torque of the knee extensor caused by different modes of RC electrical stimulation. Interested participants will then undergo an eligibility assessment by a physical therapist before the recruitment. A general medical questionnaire will be filled out by the participant as a baseline assessment that will include age, gender, BMI, and history. The collection of data will take place in the physiotherapy department lab at the University of Sharjah.

The subjects will be selected according to the following criteria: Their age ranged from 18 to 25 years, the BMI (body mass index) is within the normal range (20-25), do not have any medical conditions that have necessitated medical attention in the last two years. The subjects will be excluded from this study if they have any of the following criteria: quadriceps muscle injury spinal or lower limb fractures, lower limb deformities, abnormalities in cognition that may affect muscle and limb function, tumors or radicular symptoms, recent muscular lesions, knee injuries, sensory changes, and neuromuscular diseases; Previous surgeries that may affect the lower limb muscle performance. Any contraindication for the current Russian application. (Table1). (Table1)

TABLE 1: INCLUSION AND EXCLUSION CRITERIA FOR RC STUDY

**Inclusion criteria**	**Exclusion criteria**

• Age from 18 to 25 years<br>• BMI range 20-25<br>• Do not have any health-related condition that required medical attention in the past two years	• Quadriceps muscle injury<br>• Spinal or lower limb fractures<br>• Lower limb deformities<br>• Associated cognition abnormalities which may affect muscle and limb function.<br>• Tumors or radicular symptoms<br>• Recent muscular lesion, knee injury, sensory changes, and neuromuscular disease<br>• Previous surgery that may affect the lower limb muscle performance

Contraindications to electrical muscular stimulation listed according to guidelines “Electrophysical Agents-Contraindications and Precautions: An Evidence –Based Approach to Clinical Decision Making in Physical Therapy” by Houghton, Nussbaum, and Hoens. (Table 2) [24]

TABLE 2. SUMMARY OF ELECTRICAL STIMULATION RECOMMENDATIONS

Electrical stimulation (all forms) should not be applied

- to areas where it could cause malfunction of electronic devices, including cardiac pacemakers.
- to areas where malignancy is known or suspected.
- to people who have thrombophlebitis or active deep vein thrombosis.
- to individuals with untreated hemorrhagic diseases or to tissue that is actively bleeding.
- to infected tissues, tuberculosis, or wounds with underlying osteomyelitis.
- to recently radiated tissues
- to damaged or at-risk skin areas that would result in uneven conduction of current (excluding open wounds where the specific intent is to use electrical stimulation for tissue healing). TENS should not be applied
- to areas that have impaired sensory awareness
- NMES should not be used on pregnant women (anywhere), patients with cognitive or communication problems severe enough to prohibit them from providing accurate and timely feedback, and areas with poor circulation and any unstable area due to recent surgery, bone fracture, or osteoporosis

Electrical stimulation (all forms) can be applied with caution

- to active epiphysis
- persons with skin diseases (e.g., eczema, psoriasis)
- persons with cognition or communication impairments sufficient to prevent them from giving accurate and timely feedback e areas of impaired sensation that prevent accurate and timely feedback
- to areas with impaired circulation, if pain is not exacerbated

2.4 Study procedure and outcome measurement

Before taking part in the study, all eligible volunteers will be given information about it and asked to sign an informed consent form before the stimulation session. Baseline assessment will include demographic information, and the MVIC will be collected prior to stimulation sessions. The evaluation of primary and secondary outcomes will take place at the end of the intervention, three weeks after randomization, and at the six-week follow-up. The flow of participants throughout the investigation is represented in Fig. 2.

Primary outcome measure

Considering the potential advantages of NMES for enhancing muscle performance and the dearth of research in this area, the main objective of this study will be directed to evaluate the impact of various Russian current expert modes on the maximum electrically induced torque of the quadriceps femoris, and to compare the electrically elicited knee extensor torque at the maximal stimulation tolerance level using 3 different RC experts' modes (off mode, on mode and on2 mode).

Secondary outcome measures

The secondary objective will be to assess the degree of discomfort experienced by participants for each mode condition at the current amplitude.

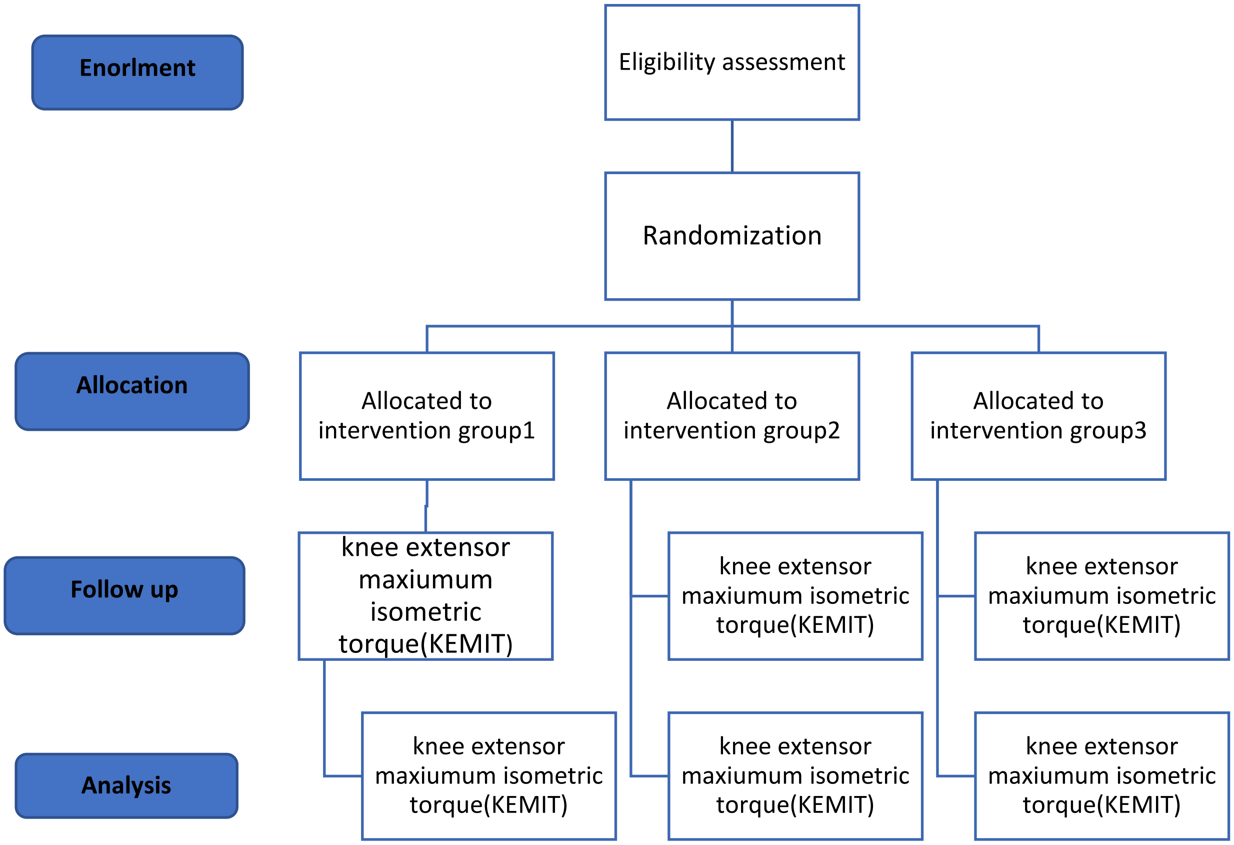

2.5 Randomization

Randomization will be used to determine which mode the participant will receive. The randomization sequence for our three groups of interest (Off mode, Rest mode, and On2 mode) will be computer-generated by one of the investigators who will not be involved in the volunteer recruiting the sequence will then be blocked (block sizes of 4, 6, and 8, in random order). The allocation will be hidden in sequentially numbered, sealed, opaque envelopes. The participants will be stratified by their gender (female or male).

2.6 Blinding

The study will include a random assignment for volunteers into three groups. The volunteers will be blinded, not aware of which electrical stimulation mode will be used. The examiner will know about the study because it requires practice and knowledge with an electrical stimulator. The statistician will also be blinded to intervention allocation. The data will be coded in an unrecognizable manner, with no information indicating which group a single participant will be assigned to.

2.7 Sample size calculation

The sample size will be based on a pilot study considering quadriceps torque as a primary outcome. The required sample size was calculated by assuming 80% power with a significance level of 0.05. To detect the minimum mean difference of 1.5 points and a standard deviation of 2.5 and considering a 10% dropout is the most suitable. [25]

2.8 Ethical consideration.

This research was approved by the Research Ethical Committee (REC)/UOS (REC-22-02-28-S) on February 28, 2022, and registered at www.clinicaltrials.gov (NCT05303181) on March 21, 2022. Consent forms will be distributed to participants prior to data collection and the general medical questionnaire. The participants will be informed that their data will be used for the study only. Privacy and confidentiality will be maintained by using a coding system for data. The data will be stored on a locked-password-required computer in the primary investigator's office and will be accessed by the study's researchers only. Participation in this study is completely voluntary. The participant has the right to leave the study at any time. This will not result in any penalty or loss of benefits to which they are entitled. If the participant decides to withdraw, any data taken from him will not be used in the research and will be destroyed.

2.9 intervention

Prior to testing, the participant's blood pressure will be taken at rest in accordance with the American College of Sports Medicine guidelines for exercise; participants will be disqualified if their resting blood pressure is greater than 180 mmHg systolic or 110 mmHg diastolic. A 5-minute warm-up on a cycle ergometer with a low-intensity warm-up will be required to reduce the possibility of muscle strain during testing. Participants then sit in a Biodex System 3 force dynamometer (Biodex Medical Systems, Inc., Shirley, NY) which will be calibrated before the session starts. To prevent excessive trunk movement, the participant will be tightly attached to the seat with three non-elastic straps: two diagonally across the chest straps from shoulder to contralateral hip and one waist strap at the level of the anterior superior iliac crests. The dynamometer will be adjusted to an angular velocity of 0°/s (isometric mode) for all tests, and the dominant limb will be in a 90° knee flexion position, which will be measured by the dynamometer's goniometer. To secure the distal leg, the lever arm of the dynamometer will be

positioned just above the ankle malleoli, while the lateral epicondyle of the knee will be in line with the axis of rotation of the lever arm. The participant's peak knee extension torque will be determined using an MVIC performed at 90° of knee flexion against the isokinetic dynamometer’s resistance arm. After being correctly seated in the Biodex device, and prior to the application of stimulation, participants will be asked to perform a minimum of three maximum voluntary isometric contractions (MVIC), The participant is required to hold each contraction for around five seconds and rest for 120 seconds (about two minutes) in between each contraction. The highest peak torque generated during the three maximum-effort trials will be called KEMVIT, where the maximum volitional peak knee extensor muscle torque (Newton meters) will be recorded using a Biodex as a baseline measurement. For instance, if there is an increase in the maximum torque by more than 5% on the third attempt, the participants will be asked to continue the contraction efforts until the increase in maximum peak torque decreases to less than 5%. The peak torque produced during the testing will be used to normalize the RC-elicited knee extensor torque to produce a percent of KEMVIT (%KEMVIT), a verbal encouragement will be given to the participants by the examiner to promote maximal effort during all contractions.

In the same position used to determine the maximum torque, RC stimulation will be administered. Two channels will be used for delivering the electrical stimulus: On the anterior thigh, four circular cutaneous electrodes with a 7.6-cm diameter will be placed. The anode will be positioned distally over the vastus medialis, with the cathode being positioned proximally over the rectus femoris. Prior to beginning testing, participants will be instructed to relax during the electrical stimulation application so that all knee extensor torque production comes directly from the electrical stimulation rather than the quadriceps muscle voluntary contraction. Participants will be monitored and instructed to self-report pain on an 11-point (0–10) numeric pain-rating scale after each

contraction, with 0 indicating "no pain" and 10 the "worst pain imaginable." Participants will be informed that a pain level of 7/10 is the maximal acceptable pain, If the pain rating reaches 7/10 or if they decide to discontinue testing for any other reason, the trials will be discontinued immediately. Similarly, the participants will be instructed to stop stimulation at a similar level of discomfort. Individuals will be submitted to the following 3 randomized test conditions, and the electrically RC elicited isometric knee extensor torque will be measured in response to each of the modes at the participants' maximum tolerance. RC stimulation session will last for 15 minutes. The Russian current neuromuscular electrical stimulation will be administered using a Chattanooga Forte 200 Combo stimulator (DJO Global, Vista, CA). The Russian currents produce 50 bursts per second at a carrier frequency of 2500 Hz, with each burst lasting 10 milliseconds and followed by a 10-millisecond inter-burst gap, resulting in a 50% duty cycle and a 200-microsecond phase duration as a fixed parameter. which automatically adjust the 3 expert modes:

OFF mode;

ON2mode; consists of two intervals the stimulus will be applied as; preset on the device, following by 2 second off and then and ON2 stimulus that has a frequency of 50 Hz and 100% amplitude.

Rest mode; consist of two intervals the stimulus will be applied as; preset on the device, then follow by a continuous Rest stimulus that has a frequency of 24 Hz and 83% amplitude.

2.10 Statistical analysis and power

For the statistical analysis of the information gathered, the SPSS 22.0 software (SPSS Statistics Ink, 176 Chicago, IL, USA) for Windows will be used. For the variables being examined,

frequency, mean, and standard deviation will be computed, as will the median and interquartile range.

The initial data will be collected from the participants before starting the program and then during the program on the 3rd and 6th weeks. For the data analysis regarding each participant, we will be using Pearson correlation (r).

The data that will be obtained from this study is a description statistic, where the mean and standard deviation of measured variables will be calculated, and inferential statistics will be used to calculate the Pearson correlation (r) to examine the EIT quadriceps muscle strength in association with the three continuous variables.

3.Discussion

3.1 Potential study impact and significance

Russian currents (medium-frequency alternating currents) are frequently utilized in rehabilitation and to enhance athletes' muscle performance, even though a lot of research has demonstrated a dose-response connection in which higher evoked muscle torques lead to stronger improvements, Analyzing NMES parameters separately can yield useful clinical data on how to effectively employ NMES to achieve strength training in people with and without injury. The expert mode is one such parameter that has been poorly studied. Electrical stimulation parameters such as phase duration, pulse duration, duty cycle, carrier frequency, and waveform type have been studied in the past. [4,14,16,25-30] However, no one addresses the effect of the RC expert mode on the quadriceps muscle in a healthy individual. The findings of this study protocol may be useful for

physiotherapists and their patients, as they could have the potential to utilize the most effective parameters when the goal is to produce maximum knee extensor torques with less discomfort.

3.2 Future research

Future research could use the protocol's findings to examine whether the results are consistent across different muscle types and patient demographics.

3.3 Study strengths and weaknesses

The current study has some limitations. The hamstring muscle group will not be explicitly observed during this procedure. The net torque of the knee extension may decrease due to the hamstrings contracting. The anterior thigh's tissue thickness makes it extremely unlikely that the current would have reached the hamstring muscles. Another limitation of this study is that this trial will only be performed on healthy volunteers' quadriceps femoris muscles. Therefore, future research will be recommended to see if the results of the current study would be similar in other muscles and patient populations.

References

1. Currier DP, Mann R. Muscular strength development by electrical stimulation in healthy individuals. Phys Ther. 1983 Jun;63(6):915-21. doi: 10.1093/ptj/63.6.915. PMID: 6856678.
2. Kubiak RJ, Whitman KM, Johnston RM. Changes in quadriceps femoris muscle strength using isometric exercise versus electrical stimulation. J Orthop Sports Phys Ther. 1987;8(11):537-41. doi: 10.2519/jospt.1987.8.11.537. PMID: 18797024.
3. Laughman RK, Youdas JW, Garrett TR, Chao EY. Strength changes in the normal quadriceps femoris muscle as a result of electrical stimulation. Phys Ther. 1983 Apr;63(4):494-9. doi: 10.1093/ptj/63.4.494. PMID: 6601279.
4. Laufer Y, Ries JD, Leininger PM, Alon G. Quadriceps femoris muscle torques and fatigue generated by neuromuscular electrical stimulation with three different waveforms. Phys Ther. 2001 Jul;81(7):1307-16. PMID: 11444994.
5. Selkowitz DM. Improvement in isometric strength of the quadriceps femoris muscle after training with electrical stimulation. Phys Ther. 1985 Feb;65(2):186-96. doi: 10.1093/ptj/65.2.186. PMID: 3871529.

6. Snyder-Mackler L, Delitto A, Stralka SW, Bailey SL. Use of electrical stimulation to enhance recovery of quadriceps femoris muscle force production in patients following anterior cruciate ligament reconstruction. Phys Ther. 1994 Oct;74(10):901-7. doi: 10.1093/ptj/74.10.901. PMID: 8090841.

7. Lai HS, Domenico GD, Strauss GR. The effect of different electro-motor stimulation training intensities on strength improvement. Aust J Physiother. 1988;34(3):151-64. doi: 10.1016/S0004-9514(14)60607-3. PMID: 25026069.

8. Carpinelli, R. N.. "THE SIZE PRINCIPLE AND A CRITICAL ANALYSIS OF THE UNSUBSTANTIATED HEAVIER-IS-BETTER RECOMMENDATION FOR RESISTANCE TRAINING." (2008).

9. Gregory CM, Bickel CS. Recruitment patterns in human skeletal muscle during electrical stimulation. Phys Ther. 2005 Apr;85(4):358-64. PMID: 15794706.

10. Bickel CS, Gregory CM, Dean JC. Motor unit recruitment during neuromuscular electrical stimulation: a critical appraisal. Eur J Appl Physiol. 2011 Oct;111(10):2399-407. doi: 10.1007/s00421-011-2128-4. Epub 2011 Aug 26. PMID: 21870119.

11. Palmieri, G. A. (1983). The principles of muscle fiber recruitment applied to strength training. In *National Strength and Conditioning Association Journal* (Vol. 5, Issue 5, p. 99).

12. Paillard T. Training Based on Electrical Stimulation Superimposed Onto Voluntary Contraction Would be Relevant Only as Part of Submaximal Contractions in Healthy Subjects. Front Physiol. 2018 Oct 12;9:1428. doi: 10.3389/fphys.2018.01428. PMID: 30369886; PMCID: PMC6194177.

13. Vanderthommen M, Duchateau J. Electrical stimulation as a modality to improve performance of the neuromuscular system. Exerc Sport Sci Rev. 2007 Oct;35(4):180-5. doi: 10.1097/jes.0b013e318156e785. PMID: 17921786.

14. McLoda TA, Carmack JA. Optimal burst duration during a facilitated quadriceps femoris contraction. J Athl Train. 2000 Apr;35(2):145-50. PMID: 16558623; PMCID: PMC1323410.

15. Snyder-Mackler L, Garrett M, Roberts M. A comparison of torque generating capabilities of three different electrical stimulating currents. J Orthop Sports Phys Ther. 1989;10(8):297-301. doi: 10.2519/jospt.1989.10.8.297. PMID: 18796954.

16. Ward AR, Robertson VJ. Variation in torque production with frequency using medium frequency alternating current. Arch Phys Med Rehabil. 1998 Nov;79(11):1399-404. doi: 10.1016/s0003-9993(98)90234-9. PMID: 9821900.

17. Stone, J. A. (1997). “Russian” Electrical Stimulation. *Athletic Therapy Today*, *2*(3), 1019–1030. https://doi.org/10.1123/att.2.3.27

18. Kramer JF, Mendryk SW. Electrical stimulation as a strength improvement technique: a review. J Orthop Sports Phys Ther. 1982;4(2):91-8. doi: 10.2519/jospt.1982.4.2.91. PMID: 18810104.

19. Akınoğlu B, Kocahan T. Russian current versus high voltage current with isokinetic training on the quadriceps muscle strength and endurance. J Exerc Rehabil. 2020 Jun 30;16(3):272-278. doi: 10.12965/jer.2040260.130. PMID: 32724785; PMCID: PMC7365730.

20. Lein DH Jr, Myers C, Bickel CS. Impact of Varying the Parameters of Stimulation of 2 Commonly Used Waveforms on Muscle Force Production and Fatigue. J Orthop Sports Phys Ther. 2015 Aug;45(8):634-41. doi: 10.2519/jospt.2015.5574. Epub 2015 Jun 24. PMID: 26107042.

21. Pinfildi CE, Andraus RAC, Iida LM, Prado RP. NEUROMUSCULAR ELECTRICAL STIMULATION OF MEDIUM AND LOW FREQUENCY ON THE QUADRICEPS FEMORIS. Acta Ortop Bras. 2018;26(5):346-349. doi: 10.1590/1413-785220182605178164. PMID: 30464720; PMCID: PMC6220661.

22. Chan AW, Tetzlaff JM, Gøtzsche PC, Altman DG, Mann H, Berlin JA, Dickersin K, Hróbjartsson A, Schulz KF, Parulekar WR, Krleza-Jeric K, Laupacis A, Moher D. SPIRIT 2013 explanation and elaboration: guidance for protocols of clinical trials. BMJ. 2013 Jan 8;346:e7586. doi: 10.1136/bmj.e7586. PMID: 23303884; PMCID: PMC3541470.

23. Eldridge SM, Chan CL, Campbell MJ, Bond CM, Hopewell S, Thabane L, Lancaster GA; PAFS consensus group. CONSORT 2010 statement: extension to randomised pilot and feasibility trials. Pilot Feasibility Stud. 2016 Oct 21;2:64. doi: 10.1186/s40814-016-0105-8. PMID: 27965879; PMCID: PMC5154046.

24. ELECTROPHYSICAL AGENTS - Contraindications And Precautions: An Evidence-Based Approach To Clinical Decision Making In Physical Therapy. Physiother Can. 2010 Fall;62(5):1-80. doi: 10.3138/ptc.62.5. Epub 2011 Jan 5. PMID: 21886384; PMCID: PMC3031347.

25. Ranjit, K. (2019). Research Methodology: A Step-by-Step Guide for Beginners - Ranjit Kumar - Google Books. In *Sage* (pp. 1–755). https://books.google.co.uk/books/about/Research_Methodology.html?id=MKGVAgAAQBAJ&redir_esc=y

26. Adams C, Scott W, Basile J, Hughes L, Leigh J, Schiller A, Walton J. Electrically Elicited Quadriceps Muscle Torque: A Comparison of 3 Waveforms. J Orthop Sports Phys Ther. 2018 Mar;48(3):217-224. doi: 10.2519/jospt.2018.7601. Epub 2017 Dec 19. PMID: 29257924.

27. Challoumas D, Biddle M, McLean M, Millar NL. Comparison of Treatments for Frozen Shoulder: A Systematic Review and Meta-analysis. JAMA Netw Open. 2020 Dec 1;3(12):e2029581. doi: 10.1001/jamanetworkopen.2020.29581. PMID: 33326025; PMCID: PMC7745103.

28. Ward AR, Oliver WG, Buccella D. Wrist extensor torque production and discomfort associated with low-frequency and burst-modulated kilohertz-frequency currents. Phys Ther. 2006 Oct;86(10):1360-7. doi: 10.2522/ptj.20050300. PMID: 17012640.

29. Lewek M, Stevens J, Snyder-Mackler L. The use of electrical stimulation to increase quadriceps femoris muscle force in an elderly patient following a total knee arthroplasty. Phys Ther. 2001 Sep;81(9):1565-71. doi: 10.1093/ptj/81.9.1565. PMID: 11688592.

30. Lai HS, Domenico GD, Strauss GR. The effect of different electro-motor stimulation training intensities on strength improvement. Aust J Physiother. 1988;34(3):151-64. doi: 10.1016/S0004-9514(14)60607-3. PMID: 25026069.
